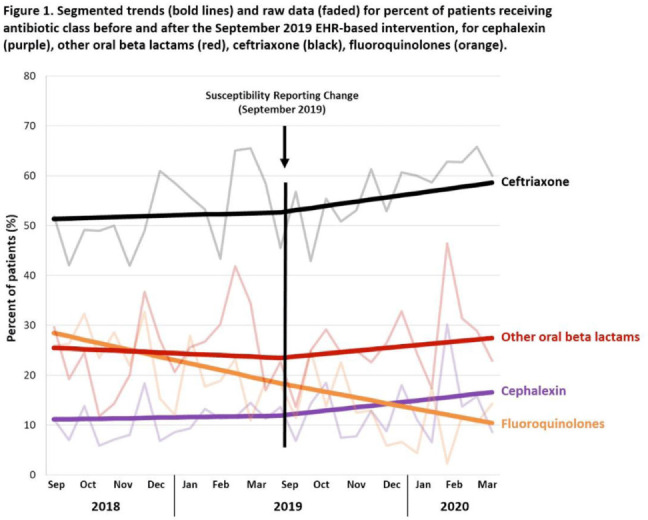# Susceptibility reporting and antibiotic prescribing for UTIs in the inpatient setting: A nudge toward improved stewardship

**DOI:** 10.1017/ash.2022.85

**Published:** 2022-05-16

**Authors:** Madison Ponder, Alan Kinlaw, Lindsay Daniels, Ashlyn Norris, Kevin Alby

## Abstract

**Background:** Urinary tract infections (UTIs) are common in the inpatient, observation, and emergency department settings. Although many UTI-causing pathogens are susceptible to oral β-lactams, these agents are not tested directly, and susceptibility is extrapolated from other agents. To improve the use of these agents, the University of North Carolina Medical Center (UNCMC) added cephalexin to the susceptibility profile generated with urine culture results in the electronic health record (EHR). We evaluated prescribing trends of cephalexin, other oral β-lactams, fluoroquinolones, and other antibiotics for UTIs in the inpatient setting, before and after the susceptibility reporting change. **Methods:** An interrupted time-series analysis was conducted. Among 1,491 patients who had positive urine cultures with susceptibilities and received at least 1 antibiotic with a listed UTI indication during their inpatient stay at UNCMC, we measured the weekly prevalence (%) of patients who received each antibiotic group: cephalexin, other oral β-lactams (amoxicillin-clavulanate, cefdinir, cefuroxime), fluoroquinolones (levofloxacin, ciprofloxacin), and ceftriaxone. The study comprised a preintervention period (September 2018–March 2019) and a postintervention period (September 2019–March 2020). The prevalence of each antibiotic or group was plotted over time, and segmented linear regression was used to estimate the impact of the intervention on each antibiotic groups’ time trend. **Results:** At study baseline in September 2018, the weekly prevalence of antibiotic use was 11% for cephalexin, 26% for other oral β-lactams, 51% for ceftriaxone, and 29% for fluoroquinolones. Fluoroquinolone use decreased steadily throughout the study period, by 11% during the 7-month preintervention period (95% CI, −17% to −5%) and by 8% (95% CI, −13% to −3%) after the intervention (*P* for trend deflection, .70). In contrast, during the preintervention period, trends were flat for cephalexin, ceftriaxone, and other oral β-lactams (all *P* for nonzero preintervention slope were >.40). During the postintervention period, use increased for ceftriaxone (6%; 95% CI, 3%–9%). Post-intervention use also increased for cephalexin (5%; 95% CI, −3% to 12%) and other oral β-lactams (4%; 95% CI, −8%, 15%), but these trends were imprecise and not statistically significant at α = .05. Fig. 1 displays trends and raw data for each antibiotic group. **Conclusions:** The urine culture susceptibility reporting change was associated with small increases in cephalexin and ceftriaxone use, coincident with continued decreasing use of fluoroquinolones, for hospitalized patients with positive urine cultures and a listed UTI indication. Low-resource EHR-based interventions may confer considerable benefit for antimicrobial stewardship efforts in this clinical setting, and larger real-world studies are needed to replicate and contextualize these findings.

**Funding:** None

**Disclosures:** None

**Figure:**